# Potential for Infra-Nodal Heart Block and Cardiogenic Shock With Propofol Administration

**DOI:** 10.4021/cr252w

**Published:** 2013-03-08

**Authors:** Nicholas Olson, Michael J. Lim, Scott W. Ferreira, Ali A. Mehdirad

**Affiliations:** a Saint Louis University, Department of Cardiology, 3635 Vista Avenue, Saint Louis, MO, 63110-0250, 13th floor Desloge Tower, 63110-0250, USA

**Keywords:** Propofol, Anesthesia, Heart block, Hypotension, Cardiogenic shock, Cardiomyopathy, Heart failure, Propofol infusion syndrome, Left ventricular assist device, Impella

## Abstract

We report a case of infra-nodal complete heart block and cardiogenic shock in a previously healthy 64-year-old man after administration of 180 mg of intravenous Propofol. Although bradycardia, hypotension, and heart block are commonly seen with propofol administration, such findings are transient and respond quickly to administration of vagolytic or sympathomimetic agents suggesting an AV nodal mechanism of heart block. Sustained left ventricular systolic dysfunction and cardiogenic shock by an alternative, non-autonomic mechanism has also been described in the setting of Propofol administration. Our case is the first to note sustained complete infra-nodal heart block in this setting. Early recognition of such a complication, restoration of atrio-ventricular (A-V) synchrony with dual chamber pacing, and aggressive circulatory support is essential in bridging such patients to recovery.

## Introduction

Propofol, a commonly utilized anesthetic agent used for conscious sedation and general anesthesia, is well known to induce bradycardia, hypotension, and heart block during its administration. Such effects are most often transient and respond quickly to the administration of vagolytic or sympathomimetic agents. Sustained left ventricular systolic dysfunction and hypotension by an alternative, non-autonomic mechanism has also been described. We report a unique case of sustained complete infra-nodal heart block and cardiogenic shock in the setting of anesthesia induction with Propofol.

## Case Report

**Figure 1:**
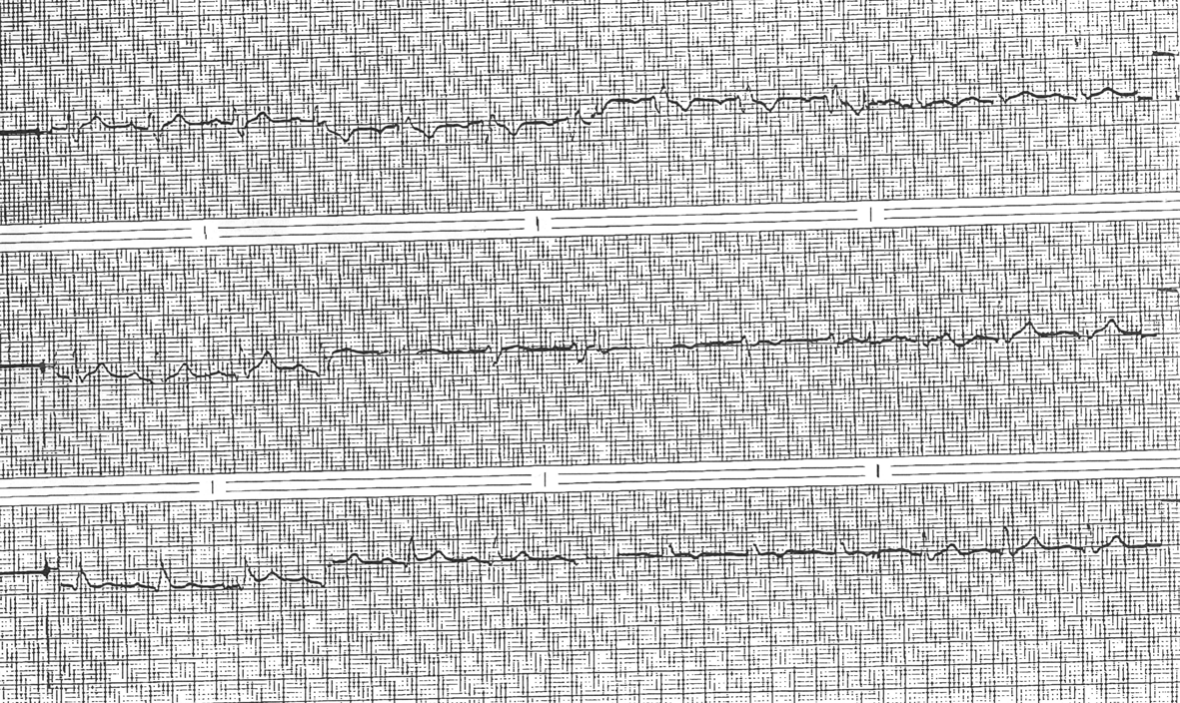
Pre-operative ECG demonstrating RBBB, PR interval of 190, and QRS duration of 150 msec.

**Figure 2:**
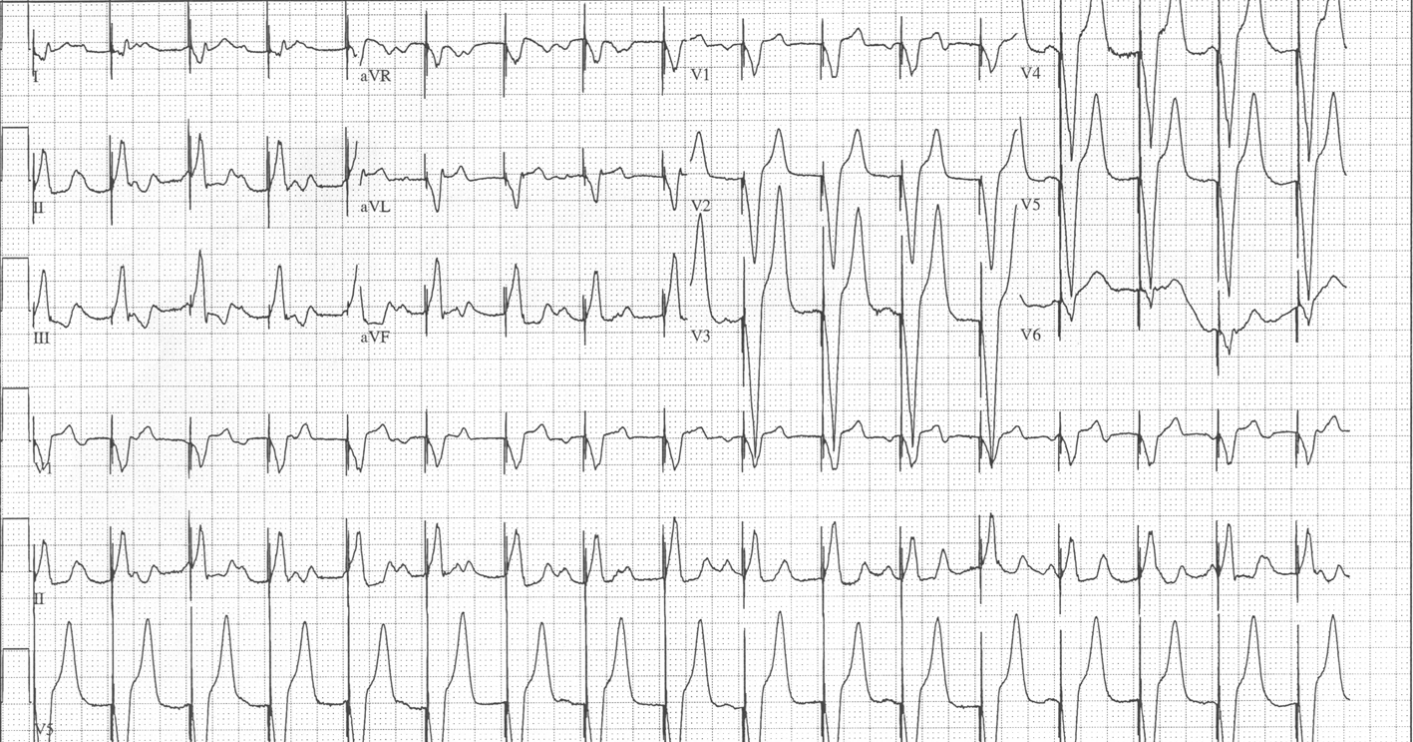
Post implantation of a single lead VVI transvenous pacemaker demonstrating sinus tachycardia at 150 bpm, complete heart block, and ventricular demand pacing at 100 bpm.

**Figure 3:**
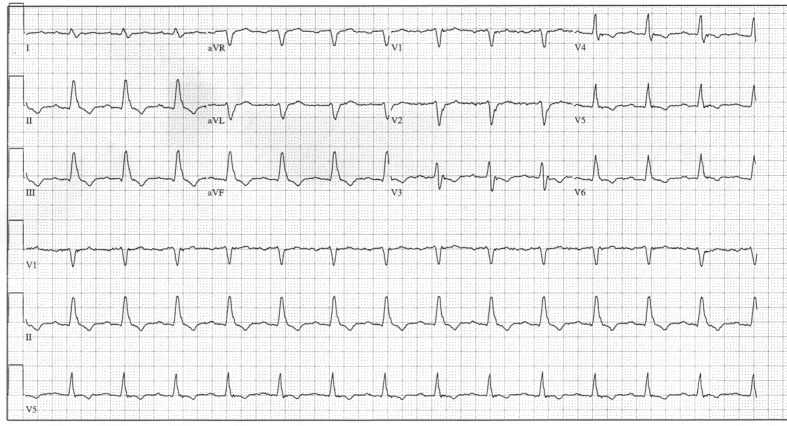
Day 5 ECG demonstrating a PR interval of 230 msec and a QRS duration of 150 msec with LBBB morphology.

**Figure 4:**
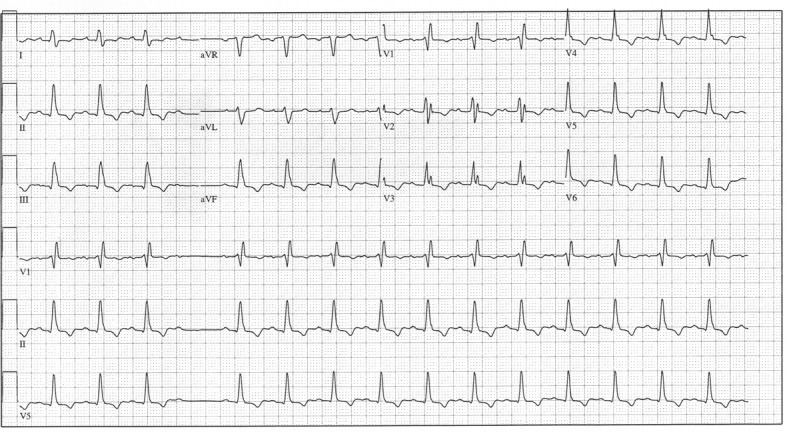
Day 5 ECG demonstrating PR interval of 190 msec and QRS duration of 150 msec with RBBB morphology and Mobitz type 2 AV block.

**Figure 5:**
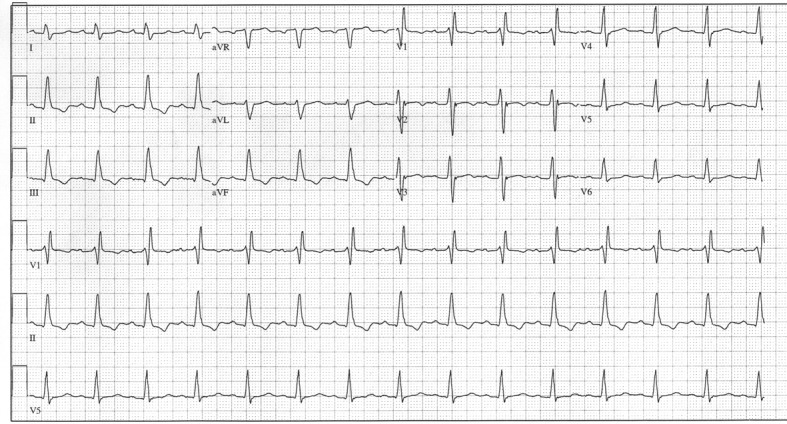
Day 6 ECG demonstrating baseline RBBB, PR interval of 188 msec and QRS duration of 150 msec.

A 64-year-old man with type II non insulin dependent diabetes mellitus presented to an outpatient surgery center for elective cataract extraction. His pre-operative ECG demonstrated sinus rhythm with a right bundle branch block (PR 190 msec and QRS duration 150 msec) (Fig. 1). Intravenous Propofol 180 mg was administered for induction of deep sedation. The patient immediately developed sinus tachycardia (sinus rate 150 bpm) with complete atrio-ventricular (AV) block. No ventricular escape rhythm was noted. The complete heart block did not respond to sequential doses of 0.5, 0.5, and 1mg of atropine. Cardiopulmonary resuscitation was initiated and the patient was orally intubated. A pulse was temporarily maintained with transcutaneous pacing and epinephrine administration during transfer to the emergency department. A temporary transvenous ventricular pacemaker was placed successfully upon arrival to the emergency department and a cutaneous hypothermic protocol was initiated. The patient continued to decompensate, developing florid pulmonary edema and increasing vasopressor requirements (Dopamine 40 µg/kg/min and Levophed 20 µg/min). A bedside transthoracic echocardiogram revealed an ejection fraction of 20%. The patient was emergently taken to the cardiac catheterization lab to ensure a non-coronary etiology and an intra-aortic balloon pump (7F Sensation, Datascope, Rastatt, Germany) was placed for additional circulatory support. Limited coronary angiography revealed no obstructive coronary artery disease. Despite continuous ventricular pacing at 100 beats/min (Fig. 2), IABP counter pulsation and escalating doses of dopamine and Levophed, the patient remained in CHB and cardiogenic shock (cardiac output 2 L/min, pulmonary capillary wedge pressure > 20 mmHg, lactate 9.7 mmol/L). On hospital day 2, continuous veno-venous hemodialysis (CVVHD) was initiated for worsening renal failure and the patient was taken back to the cardiac catheterization lab for exchange of the IABP for a left ventricular assist device (Impella 5.0, Abiomed, Danvers MA). Cardiac output increased to 3.0 L/min (Table 1). On hospital day 3, the patient continued to remain in complete heart block with no escape rhythm and was still requiring high doses of dopamine and Levophed in addition to circulatory support. In order to restore AV synchrony, a temporary transvenous atrial lead was inserted and the pacemaker mode was changed to DDD. Within 20 minutes of A-V sequential pacing at 100 bpm, the cardiac output increased to 4.8 L/min and vasopressor requirements declined over the subsequent 6 hours (Table 1). On hospital day 5, intrinsic AV conduction recovered with telemetry demonstrating an atrial-paced, ventricular-sensed rhythm. Inhibition of the transvenous pacing revealed sinus rhythm with intact AV conduction. Over the next 24 hours there were periods of both LBBB, RBBB and second degree Mobitz type 2 AV block with little change in atrial rate (Fig. 3, 4) (atrial rate 84 - 87 beats/min). The patient’s rhythm ultimately returned to baseline with a RBBB, PR interval of 188 msec, and QRS duration of 148 msec (Fig. 5). A repeat echocardiogram revealed normal LV systolic function and the left ventricular assist device was weaned. Renal function improved, CVVHD was discontinued, and the patient made a full neurological recovery. The patient declined an invasive electrophysiology study and directly underwent implantation of a permanent pacemaker prior to discharge.

**Table 1:** Hemodynamic Course

	Presentation	Post IABP^**^	Post Impella	Post A-V sequential pacer
Blood Pressure (mmHg)	80/40	70/90 Augmented	90/70	100/70
Atrial/Ventricualr Rate (beats/min)	150/100 (paced)	130/100 (paced)	130/100 (paced)	100/100 (paced)
PCWP^*^ (mmHg)	NA	28	18	15
Cardiac Output	NA	2.0 L/min	3.0 L/min	4.8 L/min
Pressor Dosages	Dopamine 40 µg/kg/min	Dopamine 40 µg/kg/min	Dopamine 40 µg/kg/min	Levophed 15µg/min
	Levophed 20 µg/min	Levophed 20 µg/min	Levophed 20 µg/min	

* PCWP: Pulmonary Capillary Wedge Pressure; ** IABP: Intra-Aortic Balloon Pump.

## Discussion

Propofol, 2, 6-diisopropylphenol, is an intravenous anesthetic that has become increasingly popular over recent years for both induction and maintenance of general anesthesia in children and adults. It has both a rapid onset of action (distribution half life of 2 - 8 min.) and a fast recovery from clinical effect (< 30 min) [1]. In addition to its sedative effect, Propofol has a number of effects on the cardiovascular system. It is well established that Propfol causes a decrease in arterial blood pressure and bradycardia at clinical concentrations. Hugg et al reviewed the hemodynamic effects of Propofol on a cohort of over 25,000 surgical patients aged 18 - 60, 15.7% developed hypotension (systolic blood pressure < 90 mmHg), 4.8% developed bradycardia (heart rate < 55 beats/min.), and 1.3% developed both [2]. Hypotension and bradycardia occurred within 10 minutes of induction in 77% and 42% of patients, respectively. Hemodynamic changes were transient and rarely required drug therapy (< 0.2% of cases).

The mechanism of these hemodynamic changes appears to be a result of decreased sympathetic tone. Ebert et al demonstrated that progressive infusions of Propofol to achieve moderate and deep sedation in healthy volunteers ages 21 - 37 resulted in significantly reduced sympathetic nerve activity, norepinephrine levels, and forearm vascular resistance [3]. These effects caused substantial decreases in mean arterial blood pressure of 9% and 18% at moderate and deep sedation, respectively. In another study by Robinson et al six volunteers were anesthetized and maintained with Propofol infusions of 125 and 200 µg/kg/min. Sympathetic denervation in the left arm was achieved with local stellate block. Forearm vascular resistance (FVR) showed no changes during Propofol infusion in the left arm (sympathetically blocked), whereas in the right arm (control) FVR decreased by 41% with both the 125 and 200 µg/kg/min infusions [4]. This strongly suggests that sympatholysis plays a large role in the mechanism of decreased systemic arterial vascular resistance.

At higher concentrations, Propofol has been shown to directly depress myocardial contractility. Sprung et al demonstrated that Propofol caused a concentration-dependent decrease in maximal myocardial tension. However such changes in myocardial contractility only became significant (P < 0.05) at supra-physiologic concentrations (> or = 56 µM). Interestingly, isoproterenol infusion restored contractility to the level prior to Propofol exposure, again suggesting a sympatholytic mechanism [5].

In addition to bradycardia and hypotension, Propofol has been commonly reported to induce transient complete heart block at clinical concentrations [6-9]. The suggested mechanism again appears to be related to a decreased sympathetic tone or hyper vagal state as the heart block predominately occurs in the setting of a slowing sinus rate and promptly resolves with atropine administration. Alphin R.S. et al examined the effects of Propofol infusion on the sinus node and atrio-ventricular node conduction properties of the guinea pig heart and demonstrated a consistent slowing of the sinus rate, prolongation of the stimulus to his (S-H) interval and prolongation of the Wenckebach cycle length and effective refractory period [10]. Atropine administration antagonized theses effects. These findings support an autonomically mediated delay in AV nodal conduction as opposed to a more ominous dysfunction of the Purkinje system itself.

In the early 1990’s a series of Propofol related deaths came to light in children who were administered high doses (7 - 10 mg/kg/hour) for prolonged periods of time (> 50 hours) [11]. The Propofol Infusion Syndrome (PRIS) as it soon became known was marked by sinus bradycardia and refractory hypotension in the setting of metabolic acidosis, rhabdomyolysis, hyperlipidemia, and hepatomegaly with intracellular fat accumulation. Similar case reports in adults soon emerged. In 2001 Cremer et al, identified 7 deaths related to unexplained metabolic acidosis and refractory hypotension in a cohort of 67 adult head injury patients who underwent high dose Propofol infusions in the neurosurgical intensive care unit [12]. Alternative mechanisms have been implicated in PRIS rather than the autonomically mediated decreases in systemic vascular resistance and reduced myocardial contractility described above. These included an impairment of mitochondrial electron transport function as well as fatty acid transport and oxidation, leading to decreased ATP production, cellular hypoxia, intracellular fatty acid accumulation and development of metabolic acidosis [13, 14].

The majority of patients in published literature with confirmed PRIS died from heart failure. However, despite a high mortality rate, PRIS has been demonstrated to be completely reversible with the cessation of Propofol and supportive measures. A report by Culp KE et al, described a case report of a 13 year old male diagnosed with PRIS after being placed on a high dose Propofol infusion (140 µg/kg/min) for cerebral edema associated with an emergent intracranial AVM repair [15]. At 72 hours of infusion, the pt. developed cardiogenic shock ultimately requiring extracorporeal circulation with membrane oxygenation (ECMO) and continuous hemodialysis. Within 48 hours, the patient’s systolic function normalized with eventual full neurological recovery.

More rarely, acute heart failure has been seen with a shorter course of low dose Propfol or an isolated bolus. Gonzalez et al described a case of a 32-year-old woman who received Propofol (1 mg/kg) and Fentanyl (100 µg) intravenously for induction of anesthesia prior to a Bartholin cyst removal [16]. Within minutes of the conclusion of the procedure, she developed vasopressor resistant cardiogenic shock eventually requiring extracorporeal membrane oxygenation. Mechanical support was weaned within 5 days and a repeat echocardiogram demonstrated normal systolic function at three weeks. Chow et al reported a case of a 19-year-old woman who was admitted for elective resection of a complex ovarian cyst [17]. Within minutes of receiving Fentanyl 100 µg IV, Midazolam 2 mg IV, and Propofol 150 mg IV she developed bradycardia, refractory hypotension and pulmonary edema. Transthoracic echocardiography revealed severe LV systolic dysfunction with an ejection fraction of 20-25%. Over the next 24 hours the patient improved without the need for mechanical support and repeat echocardiography revealed normal LV systolic function.

Our current report describes a unique case of sudden onset, long lasting complete heart block unresponsive to atropine and sympathomimetic agents accompanying cardiogenic shock with the administration of a low dose bolus of Propofol. The presence of underlying sinus tachycardia and the lack of response to vagolytic and sympathomimetic agents suggests an alternative mechanism to the atropine responsive transient heart block seen in the aforementioned case series and animal studies. Native AV conduction resumed during DDD pacing at 100 bpm (for example, the A-paced, V-paced rhythm transitioned to an A-paced, V-sensed rhythm without a change in atrial pacing rate) which is inconsistent with tachycardia dependent A-V block. The development of transient LBBB, intermittent Mobitz type 2 AVB, and ultimate transition to the patient’s baseline RBBB also occurred at a constant atrial rate (Fig. 3-5) (atrial rate 84 - 87 beats/min). This provides additional support to a recovering insult to the his-purkinje system. A pre-operative ECG demonstrating RBBB with a PR interval of 190 msec and QRS duration of 150 msec highlights pre-existing his-purkinje disease as well. In addition to the insight gained as to potential varied mechanisms of Propofol mediated CHB, our case underscores the importance of maintaining A-V synchrony during cardiogenic shock. It was only after AV sequential pacing was initiated that adequate hemodynamic support with a percutaneous LVAD was achieved (Table 1).

In conclusion, Propfol appears to have a predictable effect on the cardiovascular system resulting in a usually well tolerated and transient bradycardia, mild hypotension, and heart block at low doses. These autonomically mediated changes promptly resolve with vagolytic or sympathomimetic agents. Prolonged administration of high doses (> 100 µg/kg/hr) may impair mitochondrial function and can lead to a syndrome of a metabolic acidosis, rhabdomyolysis and severe heart failure known as the Propofol infusion syndrome. Rarely, a rapid cardiovascular decompensation with severe left ventricular systolic dysfunction, pulmonary edema, and infra-hisian complete heart block can occur with even an initial low dose bolus. Whether this represents a more rapid uncoupling of electron transport in cardiac myocyte and his-purkinje mitochondria as seen in PRIS or represents an alternative undefined mechanism remains unclear. Regardless, if Propofol is to be utilized as an anesthetic agent, a pre-operative ECG should be obtained to ascertain the presence of underlying his-purkinje disease, and if present placement of peri-operative trans-cutaneous pads and monitoring in a facility capable of rapid placement of hemodynamic support devices including an A-V sequential pacing system seems warranted. Fortunately, LV systolic and electrophysiologic recovery appears to be universal as long as such hemodynamic support is achieved.
